# Robot-Mediated Interviews - How Effective Is a Humanoid Robot as a Tool for Interviewing Young Children?

**DOI:** 10.1371/journal.pone.0059448

**Published:** 2013-03-22

**Authors:** Luke Jai Wood, Kerstin Dautenhahn, Austen Rainer, Ben Robins, Hagen Lehmann, Dag Sverre Syrdal

**Affiliations:** School of Computer Science, University of Hertfordshire, Hatfield, Hertfordshire, United Kingdom; ICREA-University of Barcelona, Spain

## Abstract

Robots have been used in a variety of education, therapy or entertainment contexts. This paper introduces the novel application of using humanoid robots for robot-mediated interviews. An experimental study examines how children’s responses towards the humanoid robot KASPAR in an interview context differ in comparison to their interaction with a human in a similar setting. Twenty-one children aged between 7 and 9 took part in this study. Each child participated in two interviews, one with an adult and one with a humanoid robot. Measures include the behavioural coding of the children’s behaviour during the interviews and questionnaire data. The questions in these interviews focused on a special event that had recently taken place in the school. The results reveal that the children interacted with KASPAR very similar to how they interacted with a human interviewer. The quantitative behaviour analysis reveal that the most notable difference between the interviews with KASPAR and the human were the duration of the interviews, the eye gaze directed towards the different interviewers, and the response time of the interviewers. These results are discussed in light of future work towards developing KASPAR as an ‘interviewer’ for young children in application areas where a robot may have advantages over a human interviewer, e.g. in police, social services, or healthcare applications.

## Introduction

In recent years, there has been a steady increase in research exploring social robots, from robotic pets and educational aids [1], [2], [3], [4], [5] to therapeutic and assistive tools for children who often respond very well to such robots [6], [7], [8], [9], [10], [11], [12].

In our previous work we have studied extensively how humans interact with robots and how robots could be designed as acceptable enjoyable, and socially intelligent interaction partners that can provide assistance to people [13]. Most relevant to this article is the minimally expressive, humanoid robot called KASPAR designed by our research group specifically for social interaction [9]. The robot has been used successfully in many human-robot interaction studies involving neurotypical [14], [15] and autistic children [10], [16], showing that children respond very well to the size and appearance of the robot and its human-like, but very simplified features. In this article, we explore a potential new application domain for KASPAR and humanoid robots in general, namely its use as a robotic interviewer for young children.

While in therapy and education the robot is typically meant to facilitate learning and/or therapeutic changes in the children, in this novel application area robots are used as mediators between a professional human interviewer and a child, providing a simple and enjoyable interaction partner with the purpose of engaging the children in the interview for the retrieval of vital information. Exploring the possibility of using robots to interview children could reveal whether robot-mediated interviews could be a valid addition to existing methods of interviewing children by professional staff such as police or social services. However, before starting investigations in the sensitive areas of interviews with children in a social services or police context, we need to establish whether or not a humanoid robot is, in more general terms, acceptable as a robotic interviewer, e.g. will children take the interviews “seriously”, i.e. discuss factual information, as opposed to treating the situation as an entertainment activity where they use their imagination? This article establishes such baseline information using a quantitative experimental approach.

Although extensive research has explored both the use of social robots with children and various approaches to interviewing children [17], [18], [19], [20], very little research investigates how robots could be used in an interview scenario. The most relevant work published by Bethel *et al.* [21] investigated if typically developing children aged 4 and 5 years old, were as likely to share a secret with a NAO robot [22], as they were with an adult. The quantitative results from this study were inconclusive. However, the qualitative results revealed that the children would readily interact with the robot and speak to it in a similar manner as they would with an adult. These results encouraged us to design a comparative experimental study to evaluate how children would respond to an interview with a robot rather than a human, and how children’s behaviour (verbally and non-verbally) may differ between the two conditions.

Note, the goal of our research is not to replace human interviewers, but to provide professionals with a robotic tool as an interface that creates an enjoyable and comfortable setting for children to talk about their experiences. Robot-mediated interviews, as described in this article, could allow the professional to precisely control the robot’s behaviour (e.g. facial expressions, body language), which is often very hard to do even for professionally trained interviewers, in particular when the topic of the interview may be emotionally sensitive.

The study was conducted in a local primary school with children aged between 7 and 9 in UK year groups 3 and 4. Each child was interviewed twice, once by a humanoid robot called KASPAR and once by a human. The interviews were counterbalanced and conducted in a structured and controlled manner in order to compare the results of the two conditions. We analysed and compared the interviews in terms of the children’s verbal and non-verbal behaviour, information disclosed during the interview, and the children’s answers to a questionnaire.

This article is structured as follows. Firstly we review literature relating to Human-Robot Interaction (HRI) and techniques for interviewing children. This is followed by a description of the structure and methodology used in our study. The findings and results from the study are then discussed. In the final section the findings and implications are assessed and the future direction of the research proposed.

## Background

### Human Robot Interaction

Scientific research investigating the use of social robots, particularly with children, has steadily increased in recent years. In this section we discuss some key contributions in this domain as relevant to this article.

Children are often more willing than adults to engage and readily interact with robots [23]. Scheeff *et al.* [24] found that young children will actively approach robots to interact with them without any instruction and that factors such as age and gender affect the interactions. This supports the hypothesis that young children may respond well to a robot interviewing them, as they are often keen to interact with robots.

Kanda *et al.* [4] conducted an 18-day field trial at a Japanese elementary school using two “Robovie” robots with first-grade and sixth-grade children to investigate the possibility of using robots as social partners to teach the children English. Although it was found that the majority of the children’s English did not improve, initially the children were very interested in the robot. Establishing a rapport with a child is essential when attempting to acquire information from them. Fior *et al.* [25] investigated if children could form relationships with robots and view them as friends. Results showed that 85.9% of the children thought the robot could be their friend, 67.4% of the children would talk to the robot, and 45.7% would share a secret with the robot. These statistics support the hypothesis that children might be willing to communicate and share information with robots as the children were happy to talk to and view robots as friends, with 45.7% of the children expressing they would be willing to share a secret with a robot.

Nishio *et al.* [26] investigated how a teleoperated android (HI-1) could be used to represent a personal presence of a real person with two young children. This research is relevant to our investigation in terms of having a robot perform conversational tasks with young children. The HI-1 robot could be deemed to fall into the uncanny valley [27], [28], and the children in Nishio’s study were both uncomfortable with the robot at first, although they did adjust to interacting with the robot. When interviewing children, which is the focus of the present article, it is important that they are as comfortable as possible with the robot from the start of the interview, in order to get the most of the interview. Therefore comparing how comfortable children are talking to a robot, as opposed to an adult, will be useful, as very little comparative research has been conducted in this specific area.

Recent work investigated how children interact with iCat robots [29], [30], [31]. Specifically the work by Shahid *et al.* [32] investigated if children perceive playing with a robot to be like playing with a friend. Results from subjective fun scores and perception tests suggested that children enjoy playing with the robot more than playing alone, but not as much as when playing with a friend. This study supports the idea that children do enjoy interacting with robots. Note, in our research, the children were always interacting with a robot or a person they had only recently met and who could be considered a stranger.

A recent closely related study by Bethel *et al.* [21] investigated if 41 children aged 4 and 5 years old, were as likely to share a secret with a NAO robot [22] as they are an adult. In this investigation a secret was shared with the child and he or she was explicitly asked not to tell anyone. Later the child took part in an interaction task with the robot and another adult separately. In the interactions the child was encouraged to tell the interaction partner the secret. The quantitative results from this study were inconclusive but the qualitative results revealed that the children would readily interact with the robot and speak to it in a similar manner as they would with an adult. Bethel et al.’s study has similarities to our research but the robot was acting as a social interaction partner rather than leading an interview. However, our investigation focused on how children would respond to a humanoid robot in a structured interview context. Also, in Bethel et al.’s study, it may not have been clear to the children that the main purpose of their interaction was to gather information from them, as they were participating in a physically interactive task. Instead, in our research it was clear to the children that the sole purpose of the interaction was for information acquisition, as there were no other tasks for them to focus on whilst having the interview.

### Interviewing Children

Social robotics and interviewing children are very different areas of research, and there has been little research investigating how robots could be used to interview children. Therefore, when exploring the possibility of using a robot to interview children, we spoke to specialist professionals from the Metropolitan Police that are experienced in interviewing young children. (The Metropolitan Police are the territorial police force responsible for Greater London and also have significant national responsibilities). These specialists advised us of how to conduct structured interviews with children, and also referred us to the Achieving Best Evidence in Criminal Proceedings document which the police refer to themselves [18], [33]. The ABE was drafted for the UK’s Home Office by a team of experts from varying backgrounds including psychology, law and social services. Because the guidelines laid out in the ABE have been well researched and recognised as providing an effective structured and standardised method for interviewing young children, we followed the relevant guidelines of the documents as closely as possible, with feedback from the above mentioned professionals during the design stages of the experiments.

The ABE suggests that interviews should have four phases in the following order:

1. Establishing a rapport
2. Asking for free narrative recall
3. Asking questions
4. Closure

This phase is used to get the child acquainted with the interviewer. The ABE suggests that this phase should be used to discuss neutral topics to relax the child and set the ground rules for the rest of the interview. In addition, the ABE states that when children have an interview with the police they instantly think that they have done something wrong and it is important to address this immediately. Although our research is not part of a criminal proceeding and the interviewers are not police officers, it was important that the children did not worry therefore we took this point into account when conducting the introduction phase as well as setting the ground rules for the interview.

When interviewing children it is desirable for the children to recall as much information as possible without prompting using minimal direction. This is because information from free narrative recall is the most accurate, and would be considered more reliable as evidence in a courtroom. Although the information the children provide in our study does not need to stand up as evidence in court, it is important for the children to express themselves freely, as we are attempting to measure how much information the children freely provide to a robot compared to a human.

The ABE suggests that once a child has recalled as much information on their own accord as possible a questioning phase should begin. In this phase the interviewer focuses on trying to recover key pieces of information that the child may have overlooked in their recall of the event. This allows the interviewer to maximise the amount of useful information they can recover from a child. The questions the police use in this phase should not be in any way leading, as this would compromise the integrity and legitimacy of the statement from the child. Although the interviews we were carrying out were not of a sensitive nature, and the statements did not need to be relied upon in court, we did include a questioning phase to maximise the information recovered and to adhere to the standardised interview structure that is used by the police. This was also useful for investigating any difference in the information the children provided to KASPAR compared to the human when asked more specific questions.

In the closing phase of the interview we followed the advice of the recommendation of the ABE, thanking the child for their time and returning to a neutral topic of conversation, similar to establishing a rapport phase.

The ABE document contains a great deal of information that is specifically useful for a police interview. In our research we followed those guidelines that were relevant to address our research questions. A lot of the criteria and information in the ABE relates to court situations and law, therefore some of the information was not applicable to our work.

### Research Questions and Expectations

This study aims to answer two general research questions that we identified as the first necessary step to establish whether or not a robot can be used as an interviewer for young children:

- How do children’s non-verbal and verbal behaviour, as well as their opinions about the interaction, differ in the two experimental conditions using a robotic versus a human interviewer? (RQ1)
- In terms of content of the children’s responses, will the children disclose more information to the robot or to the human? (RQ2)

Concerning RQ1, we expect that children will be more interested in KASPAR as a novel object [23], and would direct more behaviours that indicate interest (e.g. eye gaze) towards the robot compared to the human interviewer.

In the case of RQ2, on the one hand, one may expect that children would talk more and reveal more information to the human experimenter, since the children are very used to the situation of being asked questions by a human (e.g. at home or at school) rather than talking to a robot. On the other hand, if the children experience the robot as an enjoyable and comfortable interaction partner (compared to the human experimenter who is a stranger to them) then they might disclose more information to KASPAR. We thus expected clear preferences for either the human or robotic interviewer.

Note, both the robot and the human interviewer were presented as ‘strangers’ to the children in our experimental scenario. A novelty effect, in particular with regards to the robot, could therefore we expected, however, this reflects a natural situation of our targeted application area, where ‘strangers’ are interviewing young children once, or, if repeated, only a few times.

## Methods

### Ethics Statement

This research was approved by the University of Hertfordshire’s ethics committee for studies involving human participants. Informed consent was obtained in writing from all parents of the children participating in the study.

### Participants

The study was conducted in a local UK primary school in Hertfordshire with children aged between 7 and 9 years old (UK year groups 3 and 4). The study involved 22 children, 21 of which produced useable data, (technical difficulties meant the data from one session could not be included). Of the 21 children 10 were in year 3, 11 were in year 4, 10 were female and 11 were male. The majority of the children in year 3 were female and the majority of year 4 were male. Of the 21 children, 3 have been diagnosed with ‘some form of autism’ (according to the teachers). The results from the sessions with the children with autism were consistent with the results of the typically developing children, we therefore decided to include the data from these sessions in our dataset for this study. The adult interviewer was the first author of this article.

### Procedure

We conducted interviews with the children that took place on four days over a two-week period. Each child experienced two interviews, one with KASPAR and one with a human experimenter. The interviews were one week apart and the same interview structure was followed on both occasions. A two-phase counterbalancing method was implemented to reduce the chance of the interview order adversely influencing the results. Half of the children were interviewed by KASPAR first and half were interviewed by the human interviewer first. Counterbalancing was also applied in terms of gender and year group, so that each group had the same number of boys/girls and year groups. Assignment to each of the two groups was otherwise random for any particular child. In group 1 which were interviewed by KASPAR first there were 6 males and 5 females; the average age in this group was 8. Note, one of these females was the child not included in the final dataset because of technical difficulties. In group 2 the children were interviewed by the human first, there were 5 males and 6 females; the average age in this group was 8.3. The primary units of analysis were verbal communication, eye gaze and information disclosure.

The children were briefly given a group introduction to both KASPAR and the human interviewer at the school one day before the interviews commenced. In this introductory session we provided information on the nature of the robot (KASPAR was explicitly introduced as a robot) and on the purpose of the study (conducting interviews). It was emphasised that they were not being assessed or graded on what they did or said in the interviews and that there were no right or wrong answers. This was explained because we did not want the children to be worried or distressed about having the interview. We ensured that the children had equal minimal contact with both KASPAR and the human experimenter prior to the interviews, as having disproportionate contact could adversely affect the results. In this introduction, it was explained that we would be talking about the Red Nose Day talent event that had recently taken place, (Red Nose Day is a bi-annual national event in the UK to raise money for charities). However, the children were not provided with any details as to what they would be asked as this could lead and influence what they might have said in the interviews.

After the interviews had taken place the children were given a debrief as a group to explain how KASPAR worked. In this debrief the children were also given the opportunity to control the robot.

### The Robot

**Figure 1:**
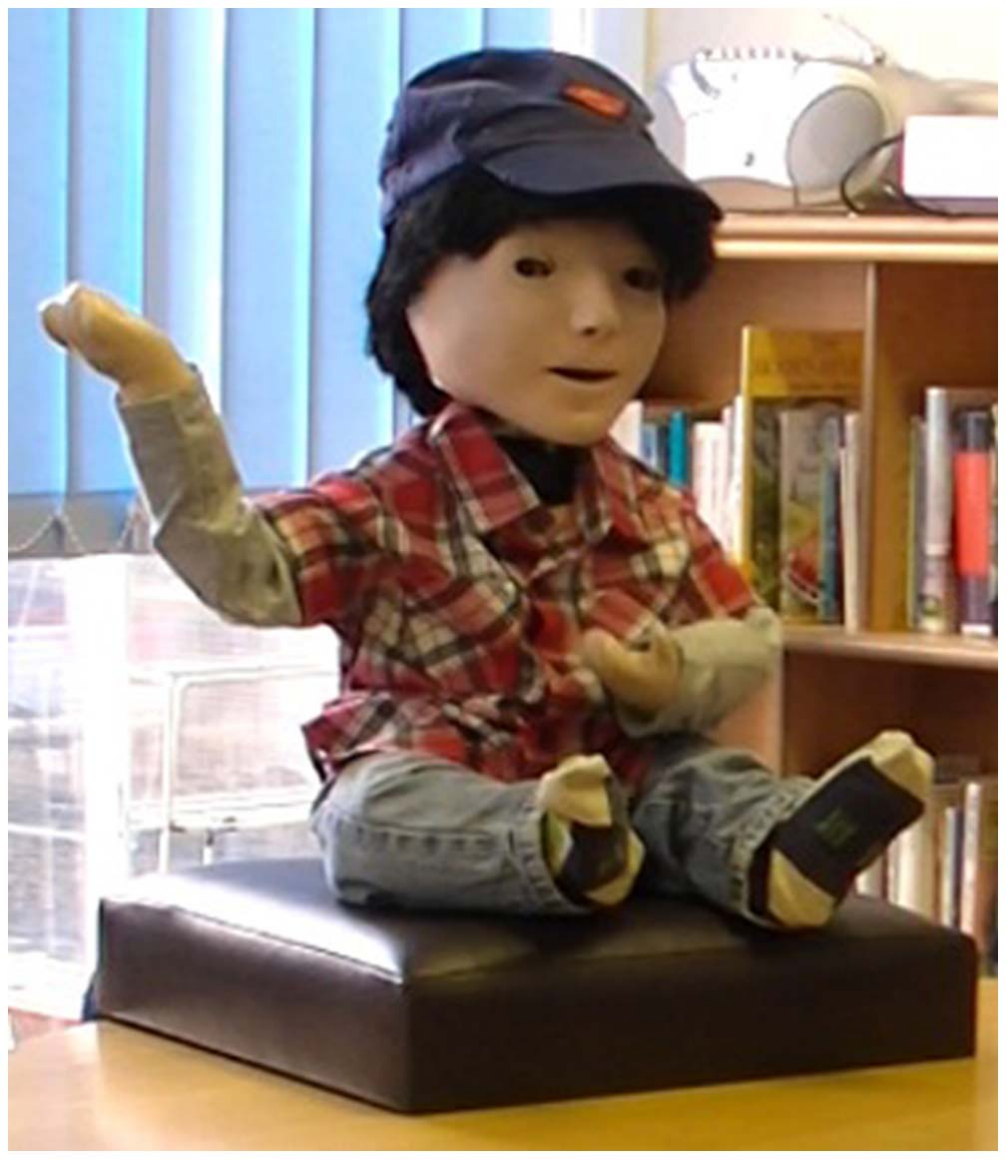
KASPAR robot.

The robot KASPAR (Figure 1) used in this study is a child-sized, humanoid robot with a minimally expressive face and arms that are capable of gesturing. This robot has been shown to be very effective when working alongside typically developing children [14], [34] and children with autism [10], [11], [16], [35]. Robins *et al.* explored how children adapt to interacting with a robot and will mirror a robot’s temporal behaviour [15]. KASPAR has also been used to explore aspects of human-robot interaction relating to the role of gestures an interaction [36], and how different types of embodiment affect interaction [14]. The previous research conducted with KASPAR would thus suggest that the robot is a suitable platform for this particular area of research investigating how children respond to a humanoid robot in an interview scenario.

The robot’s head and neck have 8 Degrees of Freedom along with the arms and hands that have 6 DOF [9]. KASPAR is controlled via a Java based GUI which can be customised for specific applications. Once setup the GUI can activate behaviours or sequences by a key press. For using the robot to conduct an interaction, speech phrases were produced by the experimenter pressing buttons, following the Wizard-of-Oz methodology (WoZ), widely used in Human-Computer Interaction (see Gould et al. 1983 [37]; Dahlback et al. 1993 [38] and more recently has been used in HRI [13], [39]. The program controlling KASPAR had been specifically tailored with pre-programmed audio clips accompanied by appropriate sequences of movements. These non-verbal or verbal behaviours (speech) were initialised by pressing specific keys, two sheets with the speech phrases and corresponding keys were in the control tent (see below) to aid the investigator in finding the correct key. The audio clips for KASPAR’s voice were generated from text-to-speech synthesis software. Text-to-speech software was used rather than recordings of a natural human voice to maintain the theme of the robot as a robot. Natural human voice coming from a clearly robotic body would most likely have impaired the perceived consistency of the robot in terms of appearance and behaviour which has been shown to be important in the human-robot interaction literature, e.g. [40], [41]. Also, using a synthesised voice helped maintain the distinction between the robot interviewer and the human interviewer. The children were unaware that KASPAR was being controlled by a human investigator. We used the WoZ methodology since in future applications that we envisage in our research, a person would speak to the child via the robot, similar to the setup used in our experiments.

### Experimental Setup

The interviews took place in a small room with a recessed portion that was mostly hidden from the children. The interviews took place in the main large area of the room at a table, while the recessed part of the room was used for the robot control tent. We used a small tent to fully hide the controls and monitor of KASPAR as the partition alone would not have fully hidden the equipment and controller. The control tent housed a small monitor with a wireless connection to camera #1 for viewing and listening to the children. This was essential as we needed to know what the children were saying in order to make KASPAR respond appropriately. Camera #1 was behind the interviewer to the left and camera #2 was also behind the interviewer and to the right, both of these cameras were recording the front of the children to capture eye gaze, while camera #3 was recording the front of the interviewer. The control tent also housed a laptop that controlled KASPAR via a remote connection.

**Figure 2:**
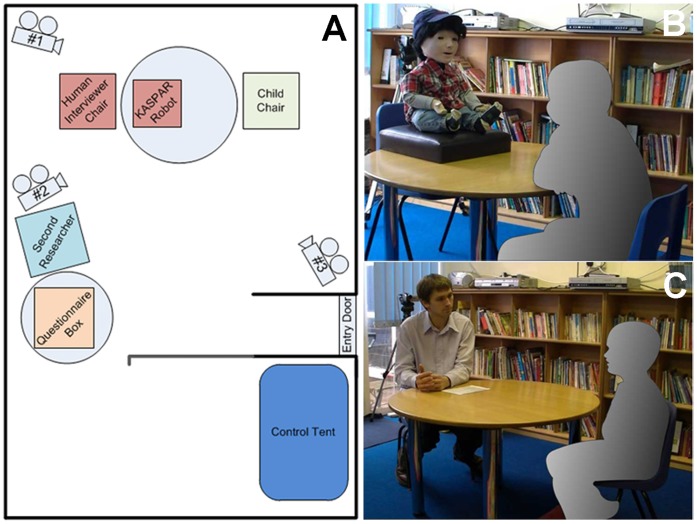
Room layout and images of scenario. (A) Room setup; (B) KASPAR interviewing a child; (C) Experimenter interviewing a child. Note the individual in this manuscript (Figure 2) has given written informed consent (as outlined in PLOS consent form) to publish these case details and photograph.

Both KASPAR and the human interviewed all the children on two separate occasions one week apart. The lead investigator always led the interview in person or remotely via KASPAR. This was important to maintain consistency, making sure that the responses and questions from both KASPAR and the human were the same. The children were taken to and from the interviews by a second researcher unknown to the children. This second researcher remained in the room during the interviews in case of any technical difficulties with the robot, but was as non-reactive as possible in order to avoid interferences with the experiment. Immediately after the interview the children were asked to complete a questionnaire and post it into a box located on a separate table. The second researcher answered any question’s the children had about the questionnaire. (Experimental setup and room layout shown in Figure 2).

### The Interview

The interviews began with a short introduction of getting to know each other’s name and ascertaining other general details such as the child’s age, if they have any siblings etc. These questions were easy for the children to answer and used to establish a rapport for the rest of the interview. We then proceeded towards the main topic, the talent event that the children had been involved in. Moving towards the main topic was achieved by asking the child “what are we going to talk about today”. If the child did not remember they were reminded that they were there to talk about the talent event. Research and practice have shown that the most detailed and reliable answers are secured from open questions [42], therefore the majority of interview questions were open questions. This maximised the children’s freedom to express themselves and minimised the scope for speculation. This approach might indicate who/what the child is more comfortable with based on how much they say and what they would say.

The questions that focused on the main topic varied in difficulty and this was reflected in the answers that the children gave. For example, the children found the question about who won the event much easier than the question about the judges who took part in the event. Almost all of the children correctly named both of the winners of the event. However, there was a much greater variation in the number of judges the children could remember. This is possibly because during the event there would have been much more focus on the winners. Also, one of the judges of the event was unfamiliar to the children and it may have been harder for them to remember the name of this individual.

The questions in this interview primarily focused on facts, similar to how the police would conduct an interview. When the police or social services are trying to gather information from an individual they are interested in the facts of an event, as it is these facts that will be used to establish what has happened before deciding on what action to take [43]. In addition to this, when making a prosecution, it is the facts and key points that are used to make the prosecution, rather than the feelings of the individual, because without the facts and points of proof a prosecution cannot be made.

The interviews concluded by thanking the child for their time and saying that it has been nice talking to them. In these interviews we adhered to a rigid structure with set sayings in order to compare the two different conditions. The majority of the structure and questions for the interview were derived from the guidance of the ABE document, specifically the “Planning and conducting interviews with children” section [18]. This provided a recognised standard approach for interviewing children. When the police are asking questions it is important to try and keep the questions as open as possible. The ABE explains that “questions beginning with the phrases ‘Tell me’, or the words ‘describe’ or ‘explain’ are useful examples of this type of question” [42], therefore we decided to use theses phrases and words at the beginning of our questions. See example questions below. (A full list of interview questions is shown in Figure S1):

- Tell me about yourself.
- Tell me what we are going to talk about today.
- Describe the event to me.
- Tell me about the judges.
- Explain what happened in the final on Friday.
- Describe for me what the winner got.

### Measurements

The questions and interview structure were reviewed and revised several times before trialling the structure, setup and equipment in the laboratory at our University with adult volunteers. The data in the school was collected from three cameras that recorded the interviews. Two of the cameras were pointing towards the front of the child from two separate angles and the other was filming behind the child and had the interviewers face in view. In addition to the interview, the children were also asked to complete a questionnaire immediately after each interview. This was to establish what they thought of the whole experience and in particular what they thought of KASPAR. The questionnaire was kept short and simple in order not to overwhelm the children. Once all the interviews were complete, the video footage was transcribed and then coded using the Observer XT software [44]. We measured verbal communication both in terms of spoken words, duration of responses and gaps between responses from the child and the interviewer. In addition, eye gaze from the child to the interviewer was coded, as well as other body language such as nodding and shaking of the head. The points of measure we used in this study were defined as follows:

#### Interview duration

Full duration of the interview from start to finish. It was used to assess if there was any difference in the time the interviews would take.

#### Eye gaze duration

This is defined as the child looking towards the interviewer’s face. We measured eye gaze duration to evaluate the different amounts of eye gaze towards the robot compared to the human. This measurement also allowed us to observe any relationship between eye gaze and the amount that the children spoke. The eye gaze measurement is proportionate to the duration of the interviews. Because the interviews varied in length it was important to take this into account.

#### Child response duration

Total amount of time the child spends speaking to the interviewer throughout the full duration of the interview.

#### Interviewer response duration

Total amount of time the interviewer spends speaking to the child throughout the full duration of the interview.

#### Response time child>interviewer

Total amount of time throughout the full duration of the interview that the interviewer takes to respond to the child.

#### Response time interviewer>child

Total amount of time throughout the full duration of the interview that the child takes to respond to the interviewer.

#### Word count

Total number of words spoken by the child throughout the full duration of the interview excluding filler words. We used this to measure how much the children spoke in each interview.

#### Filler word count

Total number of filler words spoken by the child throughout the full duration of the interview. The children would often use filler words such as “err”, “errm”, “hum”, etc. and these words were included in the transcriptions. When analysing the transcriptions for a word count these filler words were not counted in that analysis but we did perform a separate filler word analysis.

#### Proportionate word count

Total number of words spoken by the child throughout the full duration of the interview excluding filler words proportionate to the total number of words spoken by the interviewer throughout the full duration of the interview.

#### Key word count

Total number of key words spoken by the child throughout the full duration of the interview. The keywords chosen were related to the questions we asked and specifically focused on four areas:

- Family members (brothers, sisters, etc…)
- Names of judges for the talent event
- Prizes for the winners of the event
- Names of the event winners

#### Key points

In this study we also logged the key points from the transcriptions. This information consisted of 6 main categories:

- Family
- Pets
- Event acts
- Judges
- Winners
- Poster activity

The questions in our study were designed to recover information about these 6 main categories. Each category had a specific information criteria defining it as a key point. This information was analysed both qualitatively and quantitatively. The qualitative aspects are the specific details of what the children are saying and the consistency of the information between the two interviews. The quantitative aspect is the numerical logging of each specific piece of information given by the child and the statistical analysis of this logged information. The latter was done in order to understand how many key points the child revealed.

#### Key points - Family category

Since in the interviews one of the questions asked about the children’s siblings, in this category we analysed how many family members the children mention in total throughout the duration of the interview, and how many family members they state by name. We also compared the names given in both experimental conditions, to establish the consistency of the facts disclosed in both interview conditions.

#### Key points - Pets category

One of the introductory rapport building questions related to pets. Similar to the family category, we analysed for both experimental conditions how many pets the children mention and how many pets they state by name.

#### Key points - Event acts category

The questions in the interviews were designed to acquire information about the event the children either took part in or which they witnessed. In this category we logged the number of types of acts that the children mentioned, the number of acts in the event, the number of people named that took part in the event, and a comparison of the names to check consistency. With regards to the types of acts this refers to a particular sort of act i.e. dancing or singing. If a child stated a year group and an act this was also counted as a type of act because this would be a specific type of act. The number of acts in the event refers to how many acts the child stated. For example the child may have said that there were 4 types of act (dancing, singing, acting, and magic tricks) but only referred to 2 acts that were performed (the winners and the chosen act from their own year group). We also kept a record of the number of names the child mentioned who were in an act in the event.

#### Key points - Judges category

The event that the questions were based around was a talent event with judges therefore we specifically asked a question about the judges. From what the children said we establish how many judges there are in the event and also record how many they judges they name with a comparison for consistency.

#### Key points – Winner’s category

As the event was a competition there was one winning act with two children in the act and a prize. Some of the questions in the interview were designed to find out about the winner and what they received. We logged if the children could remember the winners name and if they were aware of the prize that the winners received.

#### Key points - Posters category

The children that did not take part in the main event, and only watched, made some posters to support their class mates’ acts. We logged this information and recorded how many of the children mentioned this activity because this seemed to be quite an important aspect of the event that many children mentioned.

#### Questionnaire

The children were asked to complete a questionnaire immediately after their interviews (Figure S2) to ask the children their opinions of the interview, specifically:

- Interest – How interesting they found the experience
- Difficulty – How difficult they found the interview
- Fun – How much fun they had participating
- Duration – How long they thought the interview took

## Results

We performed a series of t-tests on different measurements assessed during the experiment in order to test for statistically significant differences between the human and the robotic interviewer conditions.

**Figure 3:**
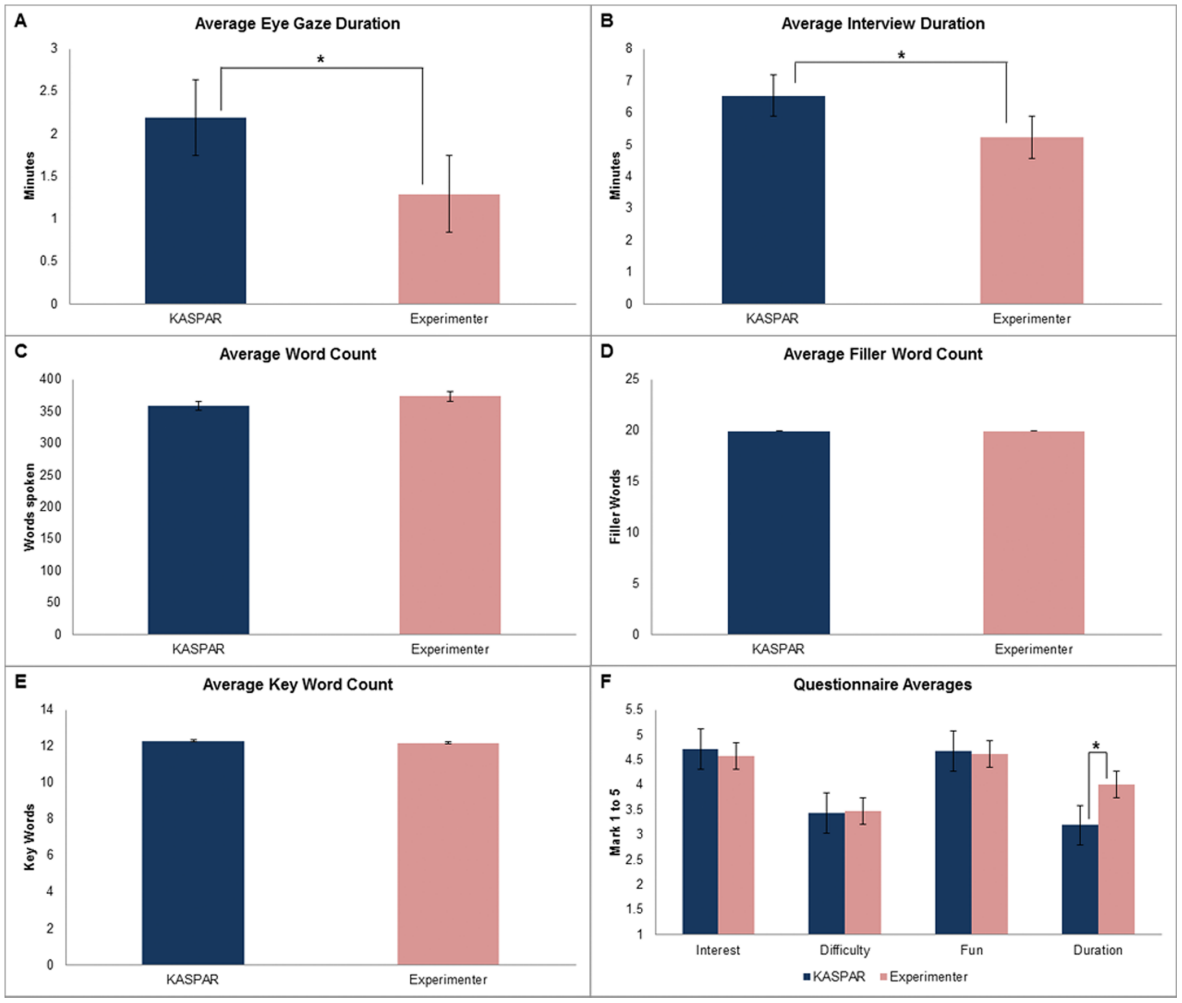
Interview averages comparison graphs. (A) Average Eye Gaze Duration; (B)Average Interview Duration; (C) Average Word Count; (D) Average Filler Word Count; (E) Average Key Word Count; (F) Questionnaire Averages.

We found that the interviews with KASPAR lasted significantly longer, on average the interviews with KASPAR lasted (minutes:seconds) 6∶53 and interviews with the human lasted 5∶22, although there was considerable variation in the durations of the interviews (see graph B in Figure 3). The interviews with KASPAR ranged from 3∶44 to 10∶45 whilst interviews with the human experimenter were between 3∶24 and 11∶43 (Table 1).

**Table 1:** Overall interaction metrics (KASPAR vs. Human).

	KASPAR		Human					
	Mean	Range	Mean	Range	Mean difference	t	p	Confidence interval of the mean
Interview duration	06∶53	3∶44–10∶45	05∶22	3∶24–11∶43	90.936	2.947	.008*	26.57–155.30
Eye gaze duration	0.338	.117–.807	0.286	.122–.717	0.053	2.115	.047*	.001–.104
Word count	359	179–672	373	175–894	−14.625	−0.415	0.683	−148
Proportionate word count	2.42	0.93–4.07	2.49	1.07–6.98	−0.074	−0.316	0.755	−0.979
Filler word count	19	2–101	19	23043	0	0	1	−11.6

Proportionately we found that the children looked towards the face of the KASPAR significantly more (Table 1). These results were normalised and calculated relative to the interview duration as the duration of the interviews varied. On average the children looked towards the face of KASPAR for 2∶19 compared to 1∶29 with the human (see graph A in Figure 3). To verify the reliability of the coding a 20% counterbalanced subset of these videos were also coded by a second independent researcher. The videos were counterbalanced in terms of interviewer, gender, year group and session. The inter-rater reliability produced a kappa value of 0.74, which is considered very good [45].

There was no significant difference in the amount of words that the children spoke to the robot compared to the human interviewer. On average the children spoke 359 words to KASPAR and to the human interviewer 373 words (see graph C in Figure 3). In addition to this there was very little difference in the amount of words the children used relative to the amount of words the interviewer used (shown under proportionate word count in Table 1).

The number of filler words the children used was very similar in both conditions (see graph D in Figure 3). On average there was no difference in the amount of filler words the children used, and in both conditions on average 19 filler words were used. However, the number filler words used with KASPAR ranged from 2 words to 101 words, and with the human experimenter from 2 words to 63 words.

There was also very little difference in the amount of keywords the children used with KASPAR compared to how many they used with the human interviewer (Table 2). On average there was less than one word difference in how many keywords were used when talking to KASPAR compared to talking to a human, with the children using approximately 12 keywords on average in both conditions (see graph E in Figure 3). The number of keywords used with KASPAR ranged from 4 words to 22 words, and with the human interviewer from 2 words to 27 words. In addition to this there was very little difference between the categories (Table 2).

**Table 2:** Key words (KASPAR vs. Human).

	KASPAR		Human					
	Mean	Range	Mean	Range	Mean difference	t	p	Confidence interval of the mean
Overall	12	4–22	12	2–27	0.095	0.122	0.904	−1.53–1.72
− Family members	4	0–12	4	0–11	0.619	1.41	0.174	−.30–1.54
− Judges names	2	0–4	2	0–2	−0.19	−0.608	0.55	−.84–.46
− Winners prizes	1	0–3	1	0–9	−0.286	−0.88	0.389	−.96–.39
− Winners names	6	0–12	6	43497	−0.048	−0.062	0.951	−1.65–1.72

In our analysis we investigated the different response durations and response times for both the child and the interviewer (Table 3). In particular we found that KASPAR took much longer to respond to the children than the human interviewer due to the technical limitations of the system. Throughout the full duration of the interviews KASPAR took an average of 1∶14 to respond to the children, while the human interviewer took an average of 20 seconds. However there is no significant difference in the time the interviewer and the child spend speaking. Therefore when calculating the children’s word count it was necessary to calculate this statistic relative to the interviewer word count. We found that proportionately the children spoke to both interviewers a similar amount relative to the interviewers word count (Table 1).

**Table 3:** Response and speaking durations (KASPAR vs. Human).

	KASPAR		Human					
	Mean	Range	Mean	Range	Mean difference	t	p	Confidence interval of the mean
Child response duration	235.3	96.28–472	220.7	97.88–618.7	14.625	0.577	0.571	−105.83
Interviewer response duration	56.2	38.6–74.2	54.42	38.24–77.16	1.803	1.131	0.271	−6.65
Response time child>interviewer	74.29	22–137.6	20	9.04–35.8	54.243	8.865	.000*	41.479–67.007
Response time interviewer>child	25.7	9–61.2	16.4	4.8–68.4	9.261	2.659	.015*	1.997–16.526

In our analysis we checked for possible effects concerning the information given by the children in relation to the questions they were asked for all of the key points categories. Firstly, we compared the number of names given by the child for each category either to the human experimenter or the robot. No significant differences were found (t = −0.36; p = 0.72). Secondly, we investigated whether the names given to each interviewer were consistent in both conditions. To check this we compared the names the children gave to just the human interviewer, just the robot interviewer and both interviewers. No significant differences in the information the children provided were found, with a mean difference of 0.33 overall. (Details of the overall statistics can be found in Table 4, whilst the details for each category are shown in Table 5).

**Table 4:** Key Points - Names listed overall (KASPAR vs. Human).

	KASPAR		Human					
	Mean	Range	Mean	Range	Mean difference	t	p	Confidence interval of the mean
All names listed total	9.67	4–21	10	3–20	0.33	−0.36	0.72	0.89
Person names listed total	1.76	0–7	1.48	0–5	0.28	0.71	0.49	0.39
Event names listed total	7.9	4–19	8.52	2–18	0.62	−0.81	0.43	0.75

**Table 5:** Key Points - Specific categories (KASPAR vs. Human).

	KASPAR		Human					
	Mean	Range	Mean	Range	Mean difference	t	p	Confidence interval of the mean
Number of family members listedby relation	2.86	1–6	2.19	0–6	0.67	1.67	0.109	0.39
Number of family members listedby name	0.9	0–6	0.62	0–3	0.28	0.95	0.36	0.29
Number of pets listed	3	0–19	5.14	0–40	2.14	−1.11	0.28	1.88
Number of pets listed by name	0.86	0–4	0.86	0–4	0	0	1	0.29
Number of types of act listed	1.67	0–9	1.67	0–7	0	0	1	0.32
Number of acts performing	3.24	1–9	3.29	1–8	0.05	−0.8	0.94	0.58
Number of performing children named	4.67	1–13	5.1	2–12	0.43	−0.7	0.51	0.63
Number of judges listed	2.52	1–5	2.33	0–6	0.19	0.64	0.53	0.29
Number of judges listed by name	1.48	0–4	1.52	0–5	0.04	−0.18	0.86	0.26
Winners prize stated	0.86	0–1	0.86	0–1	0	0	1	0
Number of winners named	1.76	0–2	1.9	0–2	0.14	−0.83	0.42	0.17
Poster activity stated	0.38	0–1	0.48	0–1	0.1	−0.81	0.43	0.11

The questionnaire results suggested that the only significant difference in how the children evaluated the interviews with KASPAR compared to the human was the average duration of the respective interviews. The children perceived that the interviews with KASPAR were longer (t = −2.364, p = 0.028*). It is also notable that the children seemed to find that the levels of difficulty talking to KASPAR or the human were similar (t = −0.204, p = 0.841), (see graph F in Figure 3).

### Order Effects

We also investigated the statistical effects of the order of the interviews. The results from this analysis revealed that there were no significant differences for the majority of measures. The only two measures (out of a total of 29 measures) where there were statistically significant differences were interviewer response duration (t = −2953, p = 0.008*) and the mention of the poster activity that the children took part in (t = 2.83, p = 0.01*). (Results of order effect analysis are shown in tables S1, S2, S3, S4, S5).

## Discussion

### Findings

The results from this study indicate that children were willing to interact with a robot in an interview scenario and did so in a similar way to how they interacted with a human interviewer. Furthermore, the amount of information that children provided to KASPAR was also very similar to the information they provided to the human. This was assessed by measuring the children’s use of keywords which we found to be similar in both the robot and human conditions. In addition, the analysis of the key points indicated that there were no significant differences in the information the children provided to KASPAR and the human interviewer. There were however statistically significant differences in both the duration of the interviews and the eye gaze toward the interviewer. The difference in the duration of the interviews can be explained by the additional time it took for the robot to respond, this was due to the technical limitations of the robot. In our data analysis we found that the robot took significantly longer to respond to the children and this is why the interviews with the robot took longer (Table 3). To confirm this we also checked by combining the time that the children spent talking, the time that the interviewer spent talking and the time that the children took responding to the robot, and this result also confirmed that the additional time taken by the interviewing the children was due to the time it took the robot to respond. Potentially this could have influenced the results of the study if this delay had caused the children to feel a disconnection in the human-robot interaction experience. However, this is not supported by our results. Note, the robot would still blink periodically during the brief periods of delays, thus maintaining the visual appearance of movement and presence of the robot.

In this study there was considerable variation in the durations of the interviews. This was due to the children all being very different in terms of how they spoke and how much information they gave. Some children were shy and would not talk much at all whilst others were very confident and would talk for a long time. Future investigations could study such individual differences in more depth, e.g. whether children’s personality traits influence their responses in interviews with a human and a robot. Previous studies have shown the influence of participants’ personality traits in human-robot interaction, e.g. [40], [46], [47].

The statistically significant difference in the durations of the interviews was due to the operation of the robot which can be confirmed from the results of the interviewer response durations (Table 3). Getting KASPAR to respond to the children takes longer than it does for a human interviewer present in the room because finding the appropriate key to respond with takes longer, despite extensive training of the operator/experimenter prior to the experiment. The results show that children looked at KASPAR more than at the human (consistent with our expectations concerning RQ1), possibly because the robot was a novel object to the children and therefore they may have been more interested in KASPAR than the human interviewing them. Ascertaining that children will respond to a robot in an interview scenario as well as to a human is an important first step in establishing that robots could be a useful tool for interviewing children.

The children’s verbal responses to were very similar in both conditions with regards to word count, filler words, key words and key points. Furthermore the children’s word count relative to the interviewers word count was similar. Both interviewers followed the same interview structure and asked the same questions. However, the interviewers are very different in terms of their nature (robot/human), so such a similarity in children’s responses in both conditions is very encouraging for developing robots as interviewing tools for children. Although the results from our study show that the children interacted with the robot in a similar manner to which they did with a human, and the information they provided is also similar, there are potential advantages a robot could have over a human interviewer. When the police are conducting interviews with children that have been through a stressful or traumatic ordeal it can be difficult for the human interviewer to maintain their composure without subtly and unintentionally indicating their thoughts and feelings. Sometimes the information that a child reveals in an interview can be quite shocking or surprising. The 2011 ABE states *“the interviewer should not display surprise at information as this could be taken as a sign that the information is incorrect”* [*48*] This can be quite difficult for a human interviewer but would be easy for a robot whose expressions are explicitly controlled, and this is one of the reasons why a robotic interviewer may have an advantage over a human interviewer in certain situations. It is also important that the interviewer does not appear to assume that someone is guilty *“So far as possible, the interview should be conducted in a ‘neutral’ atmosphere, with the interviewer taking care not to assume, or appear to assume, the guilt of an individual whose alleged conduct may be the subject of the interview”* [*49*]. Using a robot to interview a person could eliminate any of the subtle unintentional signs in body language that a human interviewer may give away, while the body language of the robot can be fully and precisely controlled by the interviewer. In addition to this the ABE states *“research shows that a person’s perceived authority can have an adverse effect on the witness, especially with respect to suggestibility”* [*50*] *.* Using a small child sized robot could potentially eliminate this problem because the robot is clearly not an adult and may not be viewed in the same way.

The children’s similar use of filler words may indicate that the children found talking to KASPAR very similar to talking to the human in terms of comfort. In some respects measuring filler words could provide a better indicator of a child’s comfort in a particular situation than a word count. The questions in the interview were focused on an event that took place on one particular day and the interviews were one week apart therefore the amount the children would remember would inevitably change. The amount of filler words the children used is likely to be more consistent with the child’s level of comfort and the number of questions asked. Some research investigating linguistic disfluencies suggests that the use of filler words could be linked to the difficulty of planning what to say [51], [52]. Whereas other research suggests that filler words my serve a communicative function to help coordinate linguistic interactions [53], for example, fillers may be used so an individual is not interrupted before they can speak their next sentence [54], [55]. There is also some evidence showing that an increased number of fillers and longer pauses occur before an uncertain answer is given [56], [57]. High disfluency has been associated with anxiety [58]. The children’s equal use of filler words in the present experiment may reflect that their comfort levels were the same with both interview partners.

Our analysis of the key points revealed that in our experiment there were no significant differences in the information the children provided to a robot compared to a human interviewer. However the analysis of the key points for each category does show that the questions in the interviews varied in difficulty. For example the children consistently named the winners of the event but often name fewer judges, even though there were more judges than winners (Table 5). This highlights that the questions in these interviews varied in difficulty.

We found no significant differences in the amount the children spoke to KASPAR, the number of keywords the children used with KASPAR, or the amount of key points the children revealed to KASPAR, compared to the human (contrary to our expectations concerning RQ2 which expected clear preferences either towards the robot or the human interviewer). However, this finding is very encouraging for the future use of robots, as it could be interpreted in such a way that children actually make no difference between human and robot interviewers in this respect and that therefore robot interviewers (i.e. robots as interviewing tools in the hands of experts remotely conducting the interview via the robot) could, with appropriate adjustments, be used as a valuable complement in interviews e.g. with social services and police.

Concerning the effect of the order of the experimental conditions, only two of the twenty-nine measures contained statistically significant differences, these were the interviewer response duration and the number of children that remembered and stated that they had taken part in a poster making activity (Tables S3, S5). It is likely that the additional time in the response duration of the interviewers is because over time the lead investigator became more comfortable and used to the interview scenario and as a result took more time responding in the later stages of the study. Although there was a statistically significant difference the mean difference is only 4.05 seconds and does not appear to have affected the interactions or the results of the study. The results of the poster activity reveal that there was a significant difference in the number of children that remembered and stated taking part in the poster making activity. The results show that more children stated taking part in a poster activity in the first phase of the interviews than the second. This is possibly because the poster activity was not the main focal point of the event and the questions in the interviews did not focus on this aspect of the event.

Generally, the findings from this study are consistent with the HRI literature as the children were happy to talk to and interact with KASPAR. The increased levels of eye gaze also suggest that the children were very interested in KASPAR. This study confirms and builds on the findings of the study by Bethel *et al.* [21] which found that children are equally likely to share a ‘secret’, or other valuable information, with a robot as they are a human. The context of the interaction and age ranges slightly differ in the two studies but the basic concept of children talking to a robot is the same.

### Limitations of Study

Concerning limitations of this study, all interviews were conducted with children attending the same school. Future work could consider schools in different geographical locations or different socio-economic status, or children with different ages. In this study we did not assess the children’s degree of introversion or extroversion. In future studies it may be useful to establish these characteristics of the child’s personality and see if this affects how the children respond to a robot compared to a human. The questions used in our interviews were based around a topic of which all the children had very different perceptions. For example some of the children took part in the audition for the event, some took part in the event, two of the children actually won the event as a pair, whilst many of the children only watched the event. This difference in perception would have affected the children’s responses although it would not have changed between the interviewers. Another limitation of the study is that the information the children were disclosing was not a ‘personal secret’ and there was no incentive for the child to keep anything from the interviewer. If the children had an invested interest in keeping information from KASPAR, or if the information had been of a more sensitive nature the results between the human and the robot may have been different. However conducting a study that focuses on questions of a personal matter could be intrusive and would be morally questionable. Our long-term goal is therefore to develop KASPAR further as a tool for practitioners, such as members of the police or social services, rather than conducting such studies ourselves. Such future studies ‘in the field’ are necessary to confirm the results obtained in the present study. The results from this study provide preliminary evidence that robots could be useful tools for interviewing children, and further investigative work needs to be carried out to confirm these results. In future studies it may also be useful to ask additional questions at the end of the interviews that could capture the children's subjective feelings about the experience of the interview with the interviewer to provide a detailed qualitative dimension. Our research has focused on a short-term one-off interview scenario rather than investigating long-term child-robot interactions. This is because our target application area is often a novel one-off situation and children generally do not have interviews on a regular basis, therefore, long-term child-robot interactions are less relevant in our target application domain. However future research could investigate the long-term effects for other potential interview applications, e.g. in a medical or educational context. If robots were to be used in these contexts it would be important to address questions such as: will the children’s behaviour differ if they are interviewed by the robot on a regular basis, and will their interest in and their co-operation with the robot decline due to the wearing off of the novelty effect?

### Summary of Hypotheses and Implications

This study investigated the difference in how children responded to a robot compared to a human in an interview scenario.

RQ1: Our expectations were supported, with the children showing significantly more eye gaze directed towards the robot’s face than the human interviewer.

RQ2: The results were contrary to our expectations. Rather than having a clear preference, the children behaved very similarly towards either of the interviewers (human/robot). The children used similar amounts of words, keywords and filler words when responding to both the robot and the human interviewer. There was also very little difference in the amount of words the children used relative to the amount of words the interviewer used. These findings illustrate that the children communicated with the robot in a similar way to which they did the human interviewer.

This study has investigated how children respond to a robot in an interview scenario compared to a human. Our results have shown that children do respond to robots in a similar way in which they respond to a human in an interview scenario. This is important because these findings can help to uncover potential advantages a robot may have over a human interviewer, for example for use by the police or social services.

### Future Work

This study provides strong support for continuing the research direction of using robots in an interview scenario with young children. Further research needs to be conducted to investigate if the responses of children vary more when they have an invested interest in keeping information from the interviewer or when they are asked questions of varying difficulty or a more sensitive nature. Our next step will be to conduct a study which will investigate how children respond to questions of varying difficulty from both a human and robotic interviewer. In addition, the capabilities of KASPAR need to be enhanced to maximise the robots potential and freedom of the interactions in terms of the ability to ask a larger variety of questions rather than pre-set questions. Apart from using robot-mediated interviews in police or social services’ investigations, other potential application areas include medical contexts (e.g. finding out about the child’s medical problems), or school contexts (e.g. when teachers try to find out details about instances involving bullying or violent behaviour). Further studies investigating robot-mediated interviews that focus on questions of a more personal and sensitive nature would need to be conducted under the expertise and guidance of a specialist interviewer.

## Supporting Information

Figure S1 Interview questions.
(PDF)

Figure S2 Questionnaire.
(PDF)

Table S1 Overall interaction metrics (Phase 1 vs. Phase 2).
(DOCX)

Table S2 Key words (Phase 1 vs. Phase 2).
(DOCX)

Table S3 Response and speaking durations (Phase 1 vs. Phase 2).
(DOCX)

Table S4 Key Points - Names listed overall (Phase 1 vs. Phase 2).
(DOCX)

Table S5 Key Points - Specific categories (Phase 1 vs. Phase 2).
(DOCX)
