# Supplementary material for: Robot-Mediated Interviews - How Effective Is a Humanoid Robot as a Tool for Interviewing Young Children?
Source: PLoS One. 2013 Mar 22;8(3):e59448. doi: 10.1371/journal.pone.0059448 (PMC3606117; doi:10.1371/journal.pone.0059448)
Supplement: Figure S1 — Interview questions. (PDF) [file pone.0059448.s001.pdf]

Interview questions

Introduction questions

- Hello, my names XXX, what's your name?
- How old are you?
- Tell me about yourself.
- Do you have any brothers or sisters?
- Tell me about them.
- Would you like any brothers or sisters?
- Do you have any pets?
- Would you like any pets?

Main interview questions

- Tell me what we are going to talk about today.
- Describe the event to me.
- Tell me what you did before the event.
- Tell me how you got to be in the event.
- Explain in detail your audition.
- Tell me about the judges.
- Explain what happened in the final on Friday.
- Tell me more about them.
- Tell me about anyone special who was there.
- Tell me all about the winner.
- Describe for me what the winner got.

Closing questions

- Did you enjoy the event?
- Well it's been nice talking to you. I'm going to meet some of your class mates now. Have fun in your lesson, and I will see you soon.
