# Supplementary material for: Robot-Mediated Interviews - How Effective Is a Humanoid Robot as a Tool for Interviewing Young Children?
Source: PLoS One. 2013 Mar 22;8(3):e59448. doi: 10.1371/journal.pone.0059448 (PMC3606117; doi:10.1371/journal.pone.0059448)
Supplement: Figure S2 — Questionnaire. (PDF) [file pone.0059448.s002.pdf]

Talking to KASPAR was?

1	2	3	4	5
Very Boring				Very Interesting

1	2	3	4	5
Very Hard				Very Easy

1	2	3	4	5
No Fun				Fun

1	2	3	4	5
Taking a long time				Very quick
