# Supplementary material for: Robot-Mediated Interviews - How Effective Is a Humanoid Robot as a Tool for Interviewing Young Children?
Source: PLoS One. 2013 Mar 22;8(3):e59448. doi: 10.1371/journal.pone.0059448 (PMC3606117; doi:10.1371/journal.pone.0059448)
Supplement: Table S1 — Overall interaction metrics (Phase 1 vs. Phase 2). (DOCX) [file pone.0059448.s003.docx]

| **Table S1. Overall interaction metrics (Phase 1 vs. Phase 2)** | | | | | | | | |
| --- | --- | --- | --- | --- | --- | --- | --- | --- |
|  | **Phase 1** | | **Phase 2** | |  |  |  |  |
|  | **Mean** | **Range** | **Mean** | **Range** | **Mean difference** | **t** | **p** | **Confidence interval of the mean** |
| Interview duration | 05:78 | 3:39 - 10:01 | 06:46 | 3:96 - 11:72 | -40.710 | -1.137 | 0.269 | 26.57 - 155.30 |
| Eye gaze duration | 0.295 | .117 - .717 | 0.329 | .124 - .807 | -0.034 | -1.291 | 0.211 | .001 - .104 |
| Word count | 339 | 175 - 543 | 394 | 179 - 894 | -54.905 | -1.642 | 0.116 | -148 |
| Proportionate word count | 2.30 | 1.07 - 3.96 | 2.61 | .98 - 6.98 | -0.302 | -1.343 | 0.194 | -0.979 |
| Filler word count | 18 | 2 - 62 | 22 | 2 - 101 | -3.714 | -1.412 | 0.173 | -11.6 |
