# Supplementary material for: Robot-Mediated Interviews - How Effective Is a Humanoid Robot as a Tool for Interviewing Young Children?
Source: PLoS One. 2013 Mar 22;8(3):e59448. doi: 10.1371/journal.pone.0059448 (PMC3606117; doi:10.1371/journal.pone.0059448)
Supplement: Table S2 — Key words (Phase 1 vs. Phase 2). (DOCX) [file pone.0059448.s004.docx]

| **Table S2. Key words (Phase 1 vs. Phase 2)** | | | | | | | | |
| --- | --- | --- | --- | --- | --- | --- | --- | --- |
|  | **Phase 1** | | **Phase 2** | |  |  |  |  |
|  | **Mean** | **Range** | **Mean** | **Range** | **Mean difference** | **t** | **p** | **Confidence interval of the mean** |
| Overall | 12 | 2 - 27 | 12 | 4 - 23 | -0.476 | -0.616 | 0.545 | -1.53 – 1.72 |
| - Family members | 4 | 0 - 11 | 4 | 0 - 12 | -0.143 | -0.311 | 0.759 | -.30 – 1.54 |
| - Judges names | 2 | 0 - 4 | 2 | 0 - 7 | 0.190 | 0.608 | 0.550 | -.84 – .46 |
| - Winners prizes | 1 | 0 - 9 | 1 | 0 - 3 | 0.476 | 1.520 | 0.144 | -.96 – .39 |
| - Winners names | 5 | 1 - 11 | 6 | 0 - 19 | -1.000 | -1.363 | 0.188 | -1.65 – 1.72 |
