# Supplementary material for: Robot-Mediated Interviews - How Effective Is a Humanoid Robot as a Tool for Interviewing Young Children?
Source: PLoS One. 2013 Mar 22;8(3):e59448. doi: 10.1371/journal.pone.0059448 (PMC3606117; doi:10.1371/journal.pone.0059448)
Supplement: Table S3 — Response and speaking durations (Phase 1 vs. Phase 2). (DOCX) [file pone.0059448.s005.docx]

| **Table S3. Response and speaking durations (Phase 1 vs. Phase 2)** | | | | | | | | |
| --- | --- | --- | --- | --- | --- | --- | --- | --- |
|  | **Phase 1** | | **Phase 2** | |  |  |  |  |
|  | **Mean** | **Range** | **Mean** | **Range** | **Mean difference** | **t** | **p** | **Confidence interval of the mean** |
| Child response duration | 209.33 | 96.28 - 361.88 | 246.66 | 98.52 - 618.68 | -37.333 | -1.544 | 0.138 | -105.83 |
| Interviewer response duration | 53.30 | 38.24 - 74.2 | 57.35 | 39.84 - 77.16 | -4.052 | -2.953 | 0.008* | -6.65 |
| Response time child > interviewer | 44.83 | 16.2 - 137.6 | 49.50 | 9.04 - 114.08 | -4.667 | -0.345 | 0.734 | 41.479 - 67.007 |
| Response time interviewer > child | 21.96 | 6.28 - 68.4 | 20.10 | 4.8 - 61.24 | 1.852 | 0.459 | 0.651 | 1.997 - 16.526 |
