# Supplementary material for: Robot-Mediated Interviews - How Effective Is a Humanoid Robot as a Tool for Interviewing Young Children?
Source: PLoS One. 2013 Mar 22;8(3):e59448. doi: 10.1371/journal.pone.0059448 (PMC3606117; doi:10.1371/journal.pone.0059448)
Supplement: Table S4 — Key Points - Names listed overall (Phase 1 vs. Phase 2). (DOCX) [file pone.0059448.s006.docx]

| **Table S4. Key Points - Names listed overall (Phase 1 vs. Phase 2)** | | | | | | | | |
| --- | --- | --- | --- | --- | --- | --- | --- | --- |
|  | **Phase 1** | | **Phase 2** | |  |  |  |  |
|  | **Mean** | **Range** | **Mean** | **Range** | **Mean difference** | **t** | **p** | **Confidence interval of the mean** |
| All names listed total | 9.52 | 3 - 21 | 10.14 | 4 - 20 | -0.62 | -0.68 | 0.50 | 0.89 |
| Person names listed total | 1.57 | 0 - 7 | 1.67 | 0 - 5 | -0.10 | -0.23 | 0.82 | 0.39 |
| Event names listed total | 7.95 | 2 - 19 | 8.48 | 2 - 21 | -0.52 | -0.68 | 0.50 | 0.75 |
