# Supplementary material for: Robot-Mediated Interviews - How Effective Is a Humanoid Robot as a Tool for Interviewing Young Children?
Source: PLoS One. 2013 Mar 22;8(3):e59448. doi: 10.1371/journal.pone.0059448 (PMC3606117; doi:10.1371/journal.pone.0059448)
Supplement: Table S5 — Key Points - Specific categories (Phase 1 vs. Phase 2). (DOCX) [file pone.0059448.s007.docx]

| **Table S5. Key Points - Specific categories (Phase 1 vs. Phase 2)** | | | | | | | | |
| --- | --- | --- | --- | --- | --- | --- | --- | --- |
|  | **Phase 1** | | **Phase 2** | |  |  |  |  |
|  | **Mean** | **Range** | **Mean** | **Range** | **Mean difference** | **t** | **p** | **Confidence interval of the mean** |
| Number of family members listed by relation | 2.48 | 1 - 6 | 2.57 | 0 - 6 | -0.10 | -0.22 | 0.82 | 0.39 |
| Number of family members listed by name | 0.86 | 0 - 6 | 0.67 | 0 - 3 | 0.19 | 0.62 | 0.54 | 0.29 |
| Number of pets listed | 2.90 | 0 - 21 | 5.24 | 0 - 40 | -2.33 | -1.22 | 0.24 | 1.88 |
| Number of pets listed by name | 0.71 | 0 - 4 | 1.00 | 0 - 4 | -0.29 | -1.00 | 0.33 | 0.29 |
| Number of types of act listed | 1.67 | 0 - 9 | 1.67 | 0 - 7 | 0.00 | 0.00 | 1.00 | 0.32 |
| Number of acts performing | 3.19 | 1 - 9 | 3.33 | 1 - 8 | -0.14 | -0.24 | 0.81 | 0.58 |
| Number of performing children named | 4.52 | 1 - 13 | 5.24 | 1 - 12 | -0.71 | -1.13 | 0.27 | 0.63 |
| Number of judges listed | 2.38 | 0 - 5 | 2.48 | 1 - 6 | -0.10 | -0.32 | 0.75 | 0.29 |
| Number of judges listed by name | 1.57 | 0 - 4 | 1.43 | 0 - 5 | 0.14 | 0.53 | 0.60 | 0.26 |
| Winners prize stated | 0.86 | 0 - 1 | 0.86 | 0 - 1 | 0.00 | 0.00 | 1.00 | 0.00 |
| Number of winners named | 1.86 | 0 - 2 | 1.81 | 0 - 2 | 0.05 | 0.27 | 0.79 | 0.17 |
| Poster activity stated | 0.57 | 0 - 1 | 0.29 | 0 - 1 | 0.29 | 2.83 | 0.01* | 0.11 |
